# Perinatal outcomes associated with low birth weight in a historical cohort

**DOI:** 10.1186/1742-4755-8-18

**Published:** 2011-06-02

**Authors:** Pedro R Coutinho, José G Cecatti, Fernanda G Surita, Maria L Costa, Sirlei S Morais

**Affiliations:** 1 Department of Obstetrics and Gynecology, School of Medical Sciences, University of Campinas, Campinas, SP, Brazil

**Keywords:** low birth weight, perinatal outcomes, prenatal care, preterm

## Abstract

**Objective:**

To identify perinatal outcomes associated with low birth weight (LBW).

**Methods:**

A retrospective cohort study in a tertiary maternity hospital. Analysis of the database on 43,499 liveborn infants delivered between 1986 and 2004 with low (n = 6,477) and normal (n = 37,467) birth weight. Outcomes associated with LBW were identified through crude and adjusted risk ratio (RR) and 95%CI with bivariate and multivariate analysis. The main outcomes were: onset of labor, mode of delivery, indication for cesarean section; amniotic fluid, fetal heart rate pattern, Apgar score, somatic gestational age, gender and congenital malformation.

**Results:**

LBW infants showed more frequently signs of perinatal compromise such as abnormal amniotic fluid volume (especially olygohydramnios), nonreassuring patterns of fetal heart rate, malformation, lower Apgar scores and lower gestational age at birth. They were associated with a greater risk of labor induction and cesarean delivery, but lower risk of forceps.

**Conclusion:**

There was a clear association between LBW and unfavorable perinatal outcomes.

## Background

Low birth weight (LBW) is a key issue in public health, especially for developing countries. It is a result of preterm delivery or the birth of a growth restricted fetus [1] and represents a major determinant of adverse health outcomes throughout life, from infancy to adulthood. Along with prematurity, it is associated with poorer indicators of child morbidity [2] and mortality [3]. There is even evidence of its association with adverse health conditions later in life, such as coronary disease, stroke, hypertension, type 2 diabetes [4] and recurrence of low birth weight in the offspring [5]. Estimates of LBW rates vary worldwide, from 3.1% to 13.3% [6]. The World Health Organization has established a goal of reducing its incidence by one third in the next decade with the objective of improving child mortality rates [7].

In the last decade increases in medically indicated labor induction and cesarean delivery have resulted in rising rates of preterm birth (PTB). In the United States alone this increase is estimated to be 45.1 per 1,000 between 1995-96 and 1999-2000 [8]. This temporal trend is also observed in developing countries. Data from Latin America show a raise in PTB due to elective induction and delivery by elective cesarean section from 10% in 1985-1990 to 18.5% in recent years [9]. This trend might be responsible for an increase in preterm deliveries, which ultimately leads to higher rates of LBW infants.

Many studies have accounted for the risk factors for preterm delivery and for LBW [6,7,10,11], as well as for neonatal outcomes. The short term outcomes on antepartum, labor and postpartum care of LBW infants have not yet been properly focused. There is no a general consensus that LBW fetuses are more susceptible to fetal distress than normal weight, that there are differences in labor, labor induction and mode of delivery between them, that newborns are prone to lower Apgar scores, and that there is a difference in gender between LBW and normal weight newborns.

Fetal heart rate monitoring is a cornerstone of antepartum surveillance in high-risk pregnancies [12]. LBW is closely associated with preterm birth and heart rate records of premature fetuses show decreased variability and little fluctuation before 28 weeks [13]. Despite that, there is no general agreement that fetuses who turn out to be LBW infants show more often non-reassuring or ominous heart rate patterns than those with normal weight. In uterus passage of meconium is also a sign of fetal compromise [2] and is associated with adverse perinatal outcomes even for preterm/very LBW newborns (birth weight ≤ 1,500 grams) [14]. Low Apgar scores at the first and the fifth minute are associated with increased risk of neurologic sequel in term infants [15]. LBW infants also present an increased risk of developing perinatal asphyxia [16]. In fact, birth weight has been shown to be independently associated with birth asphyxia [17].

The mode of delivery of infants weighting less than 1,500 grams is associated with perinatal outcomes. Cesarean is associated with increased rate of bronchopulmonary dysplasia and vaginal delivery with increased ventricular hemorrhage and higher mortality rates [18]. Vaginal breech delivery of premature infants is associated with increased neonatal mortality and morbidity (birth trauma, birth asphyxia) [19]. On the other hand, some authors suggest that cesarean is a safer route of delivery for extremely low birth weight infants [20].

It has been shown that infants with birth defects (either chromosomal or structural abnormalities) are more likely to have LBW [21]. Gender also plays a role in determining perinatal outcomes. Male fetuses are more likely to be delivered prematurely than females and show worse morbidity and mortality rates [22]. Male sex itself is considered an independent risk factor for poor pregnancy outcome [23].

In order to determine delivery and perinatal outcomes associated with LBW in the peripartum period in a cohort of 43,944 births in a tertiary public maternity in Campinas, Brazil, this current study was carried out.

## Materials and methods

A retrospective cohort study was carried out in a tertiary referral maternity hospital located in a region of 3 million inhabitants in the city of Campinas, state of São Paulo, Brazil. Information on all obstetrical hospitalizations in the institution is systematically and prospectively collected from the women's admission to their discharge. The data are reviewed and corrected if necessary by a medical supervisor prior to recording in an electronic database by a clerk.

A total of 52,136 records represent the number of deliveries at the institution between January 1986 and December 2004. Out of those records, 8,192 were excluded as they corresponded to stillbirths, twins, birth weight below 500 grams and above 4,000 grams and newborns whose birth weight was ignored. The remaining 43,944 records of livebirths were then divided into two categories: 1) newborn infants with low birth weight (< 2,500 grams) (6,477 cases; 14.7% of the population studied); and 2) newborn infants of normal weight (from 2,500 to 3,999 grams). All the 43,944 cases fulfilling the selection criteria were included in the cohort with the purpose of avoiding selection bias. A previous analysis focusing on factors associated with low birth weight was already been performed and it is published elsewhere [24].

The following delivery outcomes were considered: onset of labor (spontaneous, elective cesarean, induction), mode of delivery (vaginal cephalic, vaginal breech, forceps, cesarean), indication for cesarean (acute fetal distress, cephalopelvic disproportion, breech, other anomalous presentation, preeclampsia, previous cesarean, placental abruption, other). The perinatal outcomes considered were: characteristics of amniotic fluid (clear, meconium stained, hemorrhagic, infectious), amount of amniotic fluid (normal, olygohydramnios, polyhydramnios), fetal heart rate (normal, tachycardia, early and/or variable deceleration, late deceleration and/or bradycardia), first and fifth minute Apgar score (7-10, <7), somatic gestational age (≥37 weeks, <37 weeks), gender (male, female) and congenital malformation (with, without). For each variable with missing information, the correspondent records were excluded when statistical analysis was performed. Although it would be worth to have a differentiation between LBW due to preterm birth or fetal growth restriction, we did not consider this distinction because during the whole period there were different capacities of determining the real gestational age. In developing setting is well known the poor capacity of identifying the real gestational age, although it is believed that from one third to half of the cases of LBW were due to preterm births [25].

Using the Epi Info version 6.04b, a bivariate analysis was initially carried out where the outcomes were crossed with birth weight. The risk ratio (RR) and 95% confidence interval (95%CI) were calculated for the occurrence of outcomes comparatively between low and normal birth weight infants. These results were controlled by some confounding factors for the adjustment of the respective RR (maternal age, schooling, body mass index, smoking and time of initiation of prenatal care) using the logistic regression analysis with adjustment for RR. This was performed using the SAS software package, version 9.02. The study protocol was approved by the Institutional Review Board prior to its initiation.

## Results

The occurrence of LBW in this cohort was 14.7%. Bivariate analysis showed that with respect to labor surveillance, LBW infants were more frequently delivered after an induced labor or by elective cesarean section rather than after spontaneous onset of labor. As fetuses they showed more frequently nonreassuring or ominous heart rate patterns (tachycardia, bradycardia or late deceleration) compared with fetuses originating normal weight newborns. The association between LBW and early or variable heart rate decelerations was not significant. LBW infants were also more frequently exposed to an abnormal amniotic environment (either olygohydramnios or polyhydramnios) and hemorrhagic or infectious amniotic fluid. The occurrence of meconium stained amniotic fluid was proportionally less frequent among LBW fetuses (Table 1).

**Table 1:** Crude estimates of risks (RR and 95%CI) for labor outcomes according to birth weight

Outcomes	Low birth weight n (%)	Normal birth weight n (%)	RR (95%CI)
**Amniotic fluid (AF)**			
Clear	5,012 (77.4)	28,369 (75.7)	1.00
Meconium	834 (12.9)	7,712 (20.6)	**0.67 (0.62-0.71)**
Hemorrhagic	156 (2.4)	169 (0.5)	**5.10 (4.11-6.32)**
Infectious	123 (1.9)	108 (0.3)	**6.32 (4.89-8.16)**
Unknown	352 (5.4)	1,109 (3.0)	
**AF volume**			
Normal	5,113 (78.9)	34,297 (91.5)	1.00
Oligohydramnios	749 (11.6)	1,104 (2.9)	**4.10 (3.75-4.48)**
Polyhydramnios	147 (2.3)	444 (1.2)	**2.19 (1.82-2.63)**
Unknown	468 (7.2)	1,622 (4.3)	
**Fetal heart rate (FHR)**			
Normal	4,481 (69.2)	30,687 (81.9)	1.00
Tachycardia (>160)	124 (1.9)	273 (0.7)	**3.05 (2.48-3.77)**
Early/variable decelaration	345 (5.3)	2,164 (5.8)	1.09 (0.97-1.21)
Late decelaration/bradycardia	1,010 (15.6)	3,079 (8.2)	**2.02 (1.89-2.15)**
Unknown	517 (8.0)	1,264 (3.4)	
**Onset of labor**			
Spontaneous	3,915 (60.4)	29,282 (78.2)	1.00
Elective Cesarean section	1,580 (24.4)	3,906 (10.4)	**2.44 (2.32-2.57)**
Induction	883 (13.6)	3,834 (10.2)	**1.59 (1.49-1.70)**
Unknown	99 (1.5)	445 (1.2)	
**Total**	**6,477 (100.0)**	**37,467 (100.0)**	

With respect to the mode of delivery, there was a greater risk of LBW infants being born by cesarean (1.4 times higher) or vaginally in breech presentation (4.7 times higher), compared to cephalic vaginal delivery. On the other hand, they were protected from being delivered by forceps. For those delivered by cesarean section, placental abruption, preeclampsia and acute fetal distress appeared with a higher risk of indications for the procedure among LBW (RR 9.45, 6.04, and 2.25 times higher, respectively). Being LBW warranted protection against having a cesarean section indicated due to cephalopelvic disproportion (80%) or a repeated cesarean (14%) (Table 2).

**Table 2:** Crude estimates of risks (RR and 95%CI) for delivery outcomes according to birth weight

Outcomes	Low birth weight n (%)	Normal birth weight n (%)	RR (95%CI)
**Mode of delivery**			
Cephalic, vaginal	2,774 (42.8)	19,561 (52.2)	1.00
Breech, vaginal	126 (1.9)	181 (0.5)	**4.74 (3.79-5.93)**
Forceps	423 (6.5)	5,801 (15.5)	**0.58 (0.53-0.63)**
Cesarean	3,116 (48.1)	11,668 (31.1)	**1.42 (1.38-1.46)**
Unknown	38 (0.6)	256 (0,7)	
**Total**	**6,477 (100.0)**	**37,467 (100.0)**	
**Indication for Cesarean**			
Acute fetal distress	1,339 (43.0)	3,307 (28.3)	**2.25 (2.13-2.38)**
CPD	36 (1.2)	1,345 (11.5)	**0.20 (0.14-0.28)**
Breech	299 (9.6)	1,039 (8.9)	**1.93 (1.71-2.18)**
Preeclampsia	387 (12.4)	405 (3.5)	**6.04 (5.28-6.90)**
Previous Cesarean	328 (10.5)	2,726 (23.4)	**0.86 (0.78-0.96)**
Placental abruption	127 (4.1)	91 (0.8)	**9.45 (7.24-12.34)**
Other	600 (19.2)	2,755 (23.6)	**1.44 (1.33-1.56)**
**Total**	**3,116 (100.0)**	**11,668 (100.0)**	

LBW infants had a higher risk of being preterm, female and given Apgar scores below 7, especially at the fifth minute. They also had more frequently congenital malformation than infants of normal weight (Table 3).

**Table 3:** Crude estimates of risks (RR and 95%CI) for neonatal outcomes according to birth weight

Outcomes	Low birth weight n (%)	Normal birth weight n (%)	RR (95%CI)
**1^st ^min Apgar score**			
7-10	4,529 (70.0)	33,476 (89.3)	1.00
<7	1,681 (25.9)	3,263 (8.7)	**3.05 (2.89-3.21)**
Unknown	267 (4.1)	728 (1.9)	
**5^th ^min Apgar score**			
7-10	5,721 (88.3)	36,333 (97.0)	1.00
<7	510 (7.9)	440 (1.2)	**6.84 (6.04-7.75)**
Unknown	246 (3.8)	694 (1.8)	
**Somatic gestational age**			
≥37 weeks	2,416 (37.3)	31,971 (85.3)	1.00
<37 weeks	2,911 (45.0)	1,030 (2.7)	**17.51 (16.41-18.68)**
Unknown	1,150 (17.7)	4,466 (11.9)	
**Sex**			
Male	2,829 (43.7)	17,908 (47.8)	1.00
Female	3,009 (46.4)	16,691 (44.5)	**1.07 (1.04-1.10)**
Unknown	639 (9.9)	2,868 (7.7)	
**Malformation**			
Without	4,984 (76.9)	31,967 (85.3)	1.00
With	291 (4.5)	580 (1.5)	**3.10 (2.70-3.55)**
Unknown	1,202 (18.6)	4,920 (13.1)	
**Total**	**6,477 (100.0)**	**37,467 (100.0)**	

Multivariate analysis showed a positive association between LBW and abnormal amount of amniotic fluid and also that LBW-exposed newborns are lesser associated with non-clear AF. LBW infants showed more often as fetuses abnormal heart rate patterns during labor in comparison with fetuses that turned out to be normal weight infants. Women giving birth to LBW had also a higher risk of elective cesarean delivery or labor induction. LBW infants were at a higher risk of being delivered vaginally on breech presentation or by cesarean section, whereas with a lower risk of forceps. LBW was also independently associated with both first and fifth minutes Apgar scores <7, preterm (<37 weeks), female sex and malformation (Table 4).

**Table 4:** Adjusted risk ratio (RR_adj _and 95%CI) for maternal and perinatal outcomes from low birth weight by multiple logistic regression analysis*

Outcomes	RR_adj _(95%CI)	
**Amniotic fluid (AF)**		
clear	1.00	
non-clear	**0.79**	**(0.71-0.88)**
**AF amount**		
normal	1.00	
abnormal	**3.21**	**(2,77-3.71)**
**Fetal heart rate**		
normal	1.00	
abnormal	**1.97**	**(1.75-2.22)**
**Onset of labor**		
spontaneous	1.00	
elective cesarean	**2.27**	**(2.03-2.53)**
induction	**1.62**	**(1.43-1.84)**
**Mode of delivery**		
spontaneous	1.00	
breech	**4.69**	**(3.18-6.91)**
forceps	**0.62**	**(0.54-0.72)**
cesarean	**1.38**	**(1.29-1.48)**
**1^st ^minute Apgar score**		
7 to 10	1.00	
< 7	**2.73**	**(2.46-3.04)**
**5^th ^minute Apgar score**		
7 to 10	1.00	
< 7	**6.69**	**(5.32-8.42)**
**Somatic gestational age**		
≥ 37 weeks	1.00	
< 37 weeks	**20.80**	**(18.39-23.51)**
**Sex**		
male	1.00	
female	**1.08**	**(1.02-1.16)**
**Malformation**		
without	1.00	
with	**2.86**	**(2.24-3.64)**

* Results controlled by confounding factors: maternal age, schooling, body mass index, smoking and time of initiation of prenatal care

## Discussion

LBW infants showed more frequently signs of perinatal compromise such as abnormal amounts of AF (especially olygohydramnios), nonreassuring patterns of fetal heart rate, malformation, lower Apgar scores and lower gestational age at birth. They were associated with a greater risk of labor induction and cesarean delivery, but were protected against forceps.

In the last decades there has been a trend of a more interventionist obstetric practice. Increases in medically indicated labor induction and elective cesarean delivery have resulted in rising rates of PT birth [8], which possibly leads to higher rates of LBW infants. In this cohort LBW infants were 2.4 times more frequently delivered by cesarean section and 1.5 times more frequently delivered after labor induction than normal weight infants, what is in accordance with a more interventionist approach in the management of women presenting preterm labor or expecting growth restricted fetuses. Interestingly, there is evidence that preterm infants born after spontaneous onset of labor show lower mortality rates. It should be noted, however, that as spontaneous preterm labor is the most frequent subtype of preterm birth, it is responsible for one half of the general preterm mortality [9].

As pointed out by Goldenberg and Culhane [1], LBW is the result of either the delivery of a preterm or a growth restricted fetus. In this population it was found that LBW newborns were over 20 times more at risk of being preterm (<37 weeks) than normal weight newborns. The interpretation of fetal heart rate patterns of preterm fetuses is more difficult because of decreases in variability and little rate fluctuation before 28 weeks [13], yet fetal heart rate monitoring is still used in fetal surveillance in preterm labor. In this population LBW was associated with a greater risk of nonreassuring patterns of fetal heart rate in comparison to normal weight.

The correct determination of gestational age at birth for all cases of this population could not be completely assured. This possible limitation was the reason why de proportions of preterm and small for gestational age were not determined among the babies of this cohort. This was due to the fact that the population from this institution comes from several different referral areas and not always each patient has a good estimation of her last menstrual period or had performed an early ultrasound scan in order to have a reliable estimation of gestational age. This would probably improve the way the association with risk factors and outcomes could be analyzed.

The mode of delivery is associated with perinatal outcomes of LBW infants [19]. Over the last years cesarean section has replaced vaginal birth as the preferable and safest route of delivery of breech fetuses as the result of adverse perinatal outcomes associated with the latter [26]. This is also so for preterm and growth restricted fetuses since vaginal delivery of LBW newborns is associated with higher mortality and morbidity rates [18]. Despite that, LBW infants in this cohort were 4.7 times more frequently delivered vaginally on breech than those with normal birth weight, reflecting the fact that most of this cohort refers to a period prior to the evidence of better perinatal outcomes after cesarean sections for breech fetuses. On the other hand, they had 42% less forceps delivery than those with normal weight, what seems reasonable considering their lower weights.

Kolatat et al. [19] investigated a cohort of high risk pregnancies in a developing country and found that abnormal fetal heart rate, thick meconium and premature delivery were all risk factors for perinatal asphyxia, but only birth weight was significantly associated with this adverse outcome. In this cohort it was found that classical markers of adverse perinatal outcome, including asphyxia, such as ominous patterns of fetal heart rate, hemorrhagic or infectious amniotic fluid and lower Apgar scores were more frequently observed in LBW infants. Interestingly, LBW exposed infants seemed to be protected against meconium stained amniotic fluid, which was confirmed by the multiple regression analysis. This finding could be associated with a higher proportion of preterm among these LBW babies, but unfortunately this information was not available.

Previous studies have pointed male sex as an independent risk factor for adverse pregnancy outcome, such as premature rupture of membranes, preterm, neonatal morbidity, fetal and neonatal death [22,23]. Contrary to that evidence in this cohort LBW was associated with female sex. This might be due to the greater weight at lower gestational age of male newborns compared to females and to the fact that women expecting males have higher rates of gestational diabetes and fetal macrosomia [23]. In accordance with data from Dolan et al. [21], LBW was found to be associated with congenital malformation, as LBW infants were 3 times at risk of presenting it.

Another limitation of this study that must be addressed is the lack of reliable data on major perinatal outcomes, such as perinatal asphyxia, blood cord pH, neonatal resuscitation and neonatal morbidities. As a matter of fact those information were available in the database, but considering the long time period this population was enrolled and different sets of criteria or procedures adopted on neonatal assistance, these variables were considered not being consistently homogeneous to be evaluated.

There was a clear association between LBW and unfavorable delivery and neonatal outcomes. LBW infants showed more frequently signs of perinatal compromise (abnormal AF volume, nonreassuring patterns of fetal heart rate, malformation, lower Apgar scores and lower gestational age at birth), and were associated with a greater risk of cesarean delivery. This reinforces the importance of adequate labor surveillance in high risk pregnancies, especially women carrying growth restricted fetuses or presenting preterm labor. In fact it could be said that a more strict attention should be paid on prenatal assistance as a whole, and even more especially on neonatal care considering the current evidence that the quality of this care could make a difference in future long-term outcomes for very low birth weight infants [27].

## Disclosure of interests

The authors declare that they have no competing interests.

## Authors' contributions

JGC had the original idea for the study. PRC wrote the research protocol. PRC, JGC, FGS and MLC were responsible for data collection. SSM, PRC and JGC performed the statistical analysis. All authors saw the output of analysis drew the tables and commented on their significance. PRC wrote the first version of the manuscript, then amended and corrected by all others. All authors read the final version of the manuscript and agreed with its content before submission.

## References

[B1] Goldenberg RL Culhane JF Low birth weight in the United States Am J Clin Nutr 2007 85 S 584 90 10.1093/ajcn/85.2.584S 17284760

[B2] World Health Organization Country, regional and global estimates 2005 Geneva: WHO 54

[B3] Enkin M Keirse MJ Neilson J Crowther C Duley L Hodnett E Hofmeyr J Guide to effective care in pregnancy and childbirth 2000 3 Oxford: Oxford University Press 221 25 10.1046/j.1523-536x.2001.00041.x 11264628

[B4] Barker DJ Adult consequences of fetal growth restriction Clin Obstet Gynecol 2006 49 2 270 83 10.1097/00003081-200606000-00009 16721106

[B5] Velez MP Santos IS Matijasevich A Gigante D Gonçalves H Barros FC Victoria CG Maternal low birth weight and adverse perinatal outcomes: the 1982 Pelotas Birth Cohort Study, Brazil Rev Panam Salud Publica 2009 26 2 112 119 10.1590/S1020-49892009000800003 19814890

[B6] Nobile CG Raffaele G Altomare C Pavia M Influence of maternal and social factors as predictors of low birth weight in Italy BMC Public Health 2007 7 192 10.1186/1471-2458-7-192 17683559 PMC1959188

[B7] World Health Organization The world health report 2005. Make every mother and child count. The greatest risks to life are in its beginning 2005 Geneva: WHO 79 81

[B8] Joseph KS Theory in obstetrics: an epidemiologic framework for justifying indicated early delivery BMC Pregnancy Childbirth 2007 7 4 10.1186/1471-2393-7-4 17391525 PMC1851971

[B9] Barros FC Vélez MP Temporal trends of preterm birth subtypes and neonatal outcomes Obstet Gynecol 2006 107 5 1035 41 10.1097/01.AOG.0000215984.36989.5e 16648408

[B10] Teles EPB Faúndes A Barini R Passini R Risk factors for preterm birth in a pregnant Brazilian women sample: I. Pre-pregnant factors Rev Brasil Ginecol Obstet 1992 14 4 161 4

[B11] Vega J Saez G Smith M Agurto M Morris NM Risk factors for low birth weight and intrauterine growth retardation in Santiago, Chile Rev Med Chil 1993 121 10 1210 9 8191127

[B12] Tharmaratnam S Fetal distress Baillieres Best Pract Res Clin Obstet Gynecol 2000 14 1 155 72 10.1053/beog.1999.0069 10789266

[B13] Sweha A Hacker TW Nuovo J Interpretation of the fetal heart rate during labor Am Fam Physician 1999 59 9 2487 500 10323356

[B14] Henry JA Baker RW Yanowitz TD The in utero passage of meconium by very low birth weight infants: a marker for adverse outcomes J Perinatol 2006 26 2 125 9 10.1038/sj.jp.7211435 16407963

[B15] Asakura H Ichikawa H Nakabayashi M Ando K Kaneko K Kawabata M Tani A Satoh M Takahashi K Sakamoto S Perinatal risk factors related to neurologic outcomes of term newborns with asphyxia at birth: a prospective study J Obstet Gynaecol Res 2000 26 5 313 24 10.1111/j.1447-0756.2000.tb01333.x 11147717

[B16] Barg E Low birth weight infants (less than 2500g) - common problems for obstetricians and pediatricians Gynecol Pol 2003 74 12 1585 97 15029754

[B17] Kolatat T Vanprapar N Thitadilok W Perinatal asphyxia: multivariate analysis of risk factors J Med Assoc Thai 2000 83 9 1039 44 11075971

[B18] Munz W Seufert R Stopfkuchen H Schmidt W Pollow K Perinatal outcome of premature infants weighing less than 1500g Z Geburtshilfe Neonatol 2005 209 1 29 33 10.1055/s-2005-837797 15731978

[B19] Robilio PA Boe NM Danielsen B Gilbert WM Vaginal vs. cesarean delivery for preterm breech presentation of singleton infants in California: a population-based study J Reprod Med 2007 52 6 473 9 17694963

[B20] Barber CA Sikes NC Norton JD Lowery CL Kaiser JR Effects of mode of delivery on mortality and severe brain injury in extremely low birth weight infants in Arkansas J Ark Med Soc 2007 104 3 63 6 17902597

[B21] Dolan SM Gross SJ Merkatz IR Faber V Sullivan LM Malone FD Porter TF Nyberg DA Comstock CH Hankins GD Eddleman K Dugoff L Craigo SD Timor-Tritsch I Carr SR Wolfe HM Bianchi DW D'Alton ME The contribution of birth defects to preterm birth and low birth weight Obstet Gynecol 2007 110 2Pt1 318 24 17666606 10.1097/01.AOG.0000275264.78506.63

[B22] Ingemarsson I Gender aspects of preterm birth BJOG 2003 110 S20 34 8 10.1046/j.1471-0528.2003.00022.x 12763109

[B23] Di Renzo GC Rosati A Sarti RD Cruciani L Cutuli AM Does fetal sex affect pregnancy outcome? Gend Med 2007 4 1 19 30 10.1016/S1550-8579(07)80004-0 17584623

[B24] Coutinho PR Cecatti JG Surita FG Souza JP Morais SS Factors associated with low birth weight in a historical series of deliveries in Campinas, Brazil Rev Assoc Med Bras 2009 55 6 692 9 10.1590/S0104-42302009000600013 20191223

[B25] Barros FC Barros AJ Villar J Matijasevich A Domingues MR Victora CG How many low birthweight babies in low- and middle-income countries are preterm? Rev Saude Publica 2011 in press 10.1590/s0034-89102011005000019 21503557

[B26] Hannah ME Hannah WJ Hewson SA Hodnett ED Saigal S Willan AR Term Breech Trial Collaborative Group Planned caesarean section versus planned vaginal birth for breech presentation at term: a randomized multicentre trial Lancet 2000 356 9239 1375 83 10.1016/S0140-6736(00)02840-3 11052579

[B27] Peters KL Rosychuk RJ Hendson L Coté JJ McPherson C Tyebkhan JM Improvement of short- and long-term outcomes for very low birth weight infants: Edmonton NIDCAP trial Pediatrics 2009 124 4 1009 20 10.1542/peds.2008-3808 19786440

